# From wild to domestic and in between: how domestication and feralization changed the morphology of rabbits

**DOI:** 10.1098/rspb.2025.1150

**Published:** 2025-07-02

**Authors:** Emma Sherratt, Christine Böhmer, Cécile Callou, Thomas J. Nelson, Rishab Pillai, Irina Ruf, Thomas J. Sanger, Julia Schaar, Kévin Le Verger, Brian Kraatz, Madeleine Geiger

**Affiliations:** ^1^ School of Biological Sciences, The University of Adelaide, Adelaide, South Australia, Australia; ^2^ Zoologie und Funktionsmorphologie der Vertebraten, Zoologisches Institut, Christian-Albrechts-Universität zu Kiel, Kiel, Schleswig-Holstein, Germany; ^3^ Bioarchéologie, Interactions Sociétés Environnement (BioArch), Muséum National d'Histoire Naturelle, CNRS, Paris, France; ^4^ Abteilung Messelforschung und Mammalogie, Senckenberg Forschungsinstitut und Naturmuseum Frankfurt, Frankfurt am Main, Germany; ^5^ Institut für Geowissenschaften, Goethe-Universität Frankfurt am Main, Frankfurt am Main, Germany; ^6^ Research Center of Paleontology and Stratigraphy, Jilin University, Changchun, China; ^7^ Department of Biology, Loyola University Chicago, Chicago, IL, USA; ^8^ Department of Paleontology, University of Zurich, Zurich, Switzerland; ^9^ Department of Anatomy, Western University of Health Sciences, Pomona, CA, USA; ^10^ Natural History Museum St Gallen, St Gallen, Switzerland; ^11^ SWILD – Urban Ecology & Wildlife Research, Zurich, Switzerland

**Keywords:** domestication, feralization, allometry, *Oryctolagus cuniculus*, invasive species, morphometrics

## Abstract

One of the world’s most recognizable domestic animals is the rabbit, domesticated from the European rabbit. Domestication can drive morphospace expansion into novel phenotypes not observed in their wild counterparts; the consequences of feralization are less understood. Through these processes, we examine how humans have inadvertently driven great morphological change in this species. Characterizing skull morphological variation in a global sample of 912 wild, feral and domesticated specimens, we show that both domestication and feralization of rabbits have resulted in novel morphologies that leverage evolutionary patterns ubiquitous in the leporid clade: allometry and facial tilt are the principal axes of variation in rabbits as seen across species of Leporidae. However*,* rabbits diversified into regions of morphospace not explored by other species of their family. Although feralization of rabbits cannot be regarded as a ‘reversal’ to the wild form, we found they occupy an intermediate position, as well as their own allometric trajectory. Such insights from human-induced and environmentally driven morphological change of domestics and feral animals, respectively, reveal how novel morphologies can evolve at the macroevolutionary level. Future studies may elucidate the evolutionary, functional and developmental drivers and constraints of the cranial patterns observed in the rabbit.

## 1. Introduction

Domestication can potentially drive increased phenotypic variation expanding the morphospaces with morphologies as yet unexplored by their wild counterparts. Therefore, domesticated animals have proven important in understanding the origins of biological diversity. Darwin recognized this potential in volume 1 of his 1868 book *The Variation of Animals and Plants under Domestication* [1]. In recent years, there has been a surge of interest in characterizing variation in domestic breeds relative to their wild counterparts to understand the influence domestication has on disparity (e.g. [2–10]).

Predictable morphological changes associated with domestication are called ‘domestication syndrome’ [1,11] (reviewed in [12]). Among the commonly observed morphologies, the skull is expected to have a proportionally shorter face and smaller brain (e.g. [13]). However, these trait transformations are not pervasive [12,14]; only certain, highly derived modern breeds have been found to comply [12] (but see [15]). Feralization is the process by which domestic animals become established *in absentia* of purposeful anthropogenic propagation [16]. Historically viewed as a reversal of domestication, it is usually studied to understand how permanent domestication traits are for the phenotype (e.g. [17–20]). While one may expect that feral animals will revert towards wild morphologies with subsequent generations, the limited evidence is mixed, and so it may be argued that these are not opposite processes [16,21,22].

One of the world’s most recognizable domestic animals is the rabbit, which is domesticated from the European rabbit (*Oryctolagus cuniculus*)—purportedly by French monks during the Middle Ages [23,24]. Today, rabbits are distributed across the world, in free-ranging populations resulting from human introductions, as pets and laboratory research models, and they are also farmed for consumption and fur [25]. Notably, they have been ecologically disastrous as invasive species in Australia [26], Chile [27] and Argentina [28], as well as numerous Pacific islands [29,30]. Yet they are a ‘conservation paradox’, being endangered in their native range of the Iberian Peninsula in western Europe [31]. Through domestication, and subsequent feralization of domesticated rabbits moving into wild ecosystems, humans have inadvertently driven great morphological change in this species. This allows researchers to address one of the fundamental questions of evolutionary biology: how does morphological diversity arise? Digital imaging in the form of laser surface scans and X-ray computed tomography (CT), and data sharing, have presented the first opportunity to quantify the amount of skeletal variation encompassed by rabbits from wild, domestic and feral populations around the world, describe the primary ways they have changed and thus infer the biological processes underlying this diversity.

In modern-day Europe, two subspecies of *O. cuniculus* are recognized in their native range in the Iberian Peninsula: *O. c. cuniculus* and *O. c. algirus*. Palaeontological studies have documented the morphological and species diversity of the genus *Oryctolagus* across Europe during the Pleistocene [32,33]. Modern population variation in wild *O. cuniculus* morphology has been studied in several restricted geographical areas, such as the Maltese archipelago [34], Sicily/Italy [35], Las Lomas/Spain [36], and in broader areas such as Australia [37–40], western Europe and North Africa [24,41,42]. Variation among the approximately 200 modern breeds of domestic rabbits is extensive [43] and was described in detail by Darwin [1]. Rabbit breeds and their phenotypic diversity have also been quantified more recently [3,44,45]. Body size variation is an important finding in many of these studies, with wild rabbits in Europe generally following Bergmann’s rule [24,42], and domesticated rabbit breeds showing great allometric diversity [3,44]. Size variation is also reported to underlie morphological diversity patterns observed across different ecological regions of Australia [38–40].

The European rabbit is just one member of an overlooked ecologically diverse radiation, the family Leporidae [25]. The leporid skull has proved to exhibit great adaptive scope informing on behavioural, sensory and even locomotive evolution of the family [46–49] and can be used as a proxy for body size [50]. This study aims to characterize the morphological disparity of the European rabbit skull in wild, feral and domestic animals sampled globally, and contrast this disparity with the Leporidae radiation. We sampled 912 rabbit specimens, using natural history museum collections and invasive species control programs. We included specimens representing the wild form of the species from its contemporary native range in Spain, Portugal and southwestern France, independent wild/feral populations and domestic animals from 20 different worldwide locations (countries, territories, islands). Applying geometric morphometric methods to quantify shape and size variation in the skull, we assessed size-related (allometric) shape diversity that this species has acquired through several hundred years of domestication and feralization. We examined whether domestic rabbits have predictable skull proportions—relatively shorter face length and smaller braincase size—as hypothesized under ‘domestication syndrome’, and if feralization has resulted in a reversion to the wild form. Finally, we used an existing dataset of 24 leporid species encompassing all 11 modern genera [47,51] to provide an evolutionary baseline of morphological disparity with which to compare the wild, feral and domesticated rabbit.

## 2. Material and methods

### (a) Samples

We considered three broad categories of rabbits: ‘domesticated’, which included pets, farmed and laboratory animals; ‘wild’, being free-ranging rabbits exclusively from the native range of *O. cuniculus* in the Iberian Peninsula; and ‘feral’, being free-ranging rabbits outside of this native region, including those introduced from domesticated (usually farmed) stock as well as likely natural migration events. Natural migration events were included in the feral group because such migrations are usually human-aided and incorporate populations that represent stages on the domestication continuum (see [3], and references therein).

A total of 912 specimens of *O. cuniculus* were sourced from natural history museum collections, mostly stored as dry skeletons or as alcohol-preserved specimens (electronic supplementary material, table S1). The wild-type *Oryctolagus* from Portugal, Spain and Southwestern France cover both subspecies, although the exact taxonomic assignment is not available for all specimens. In Australia, whole carcasses resulting from pest control activities were also obtained (details of permits and collection in electronic supplementary material, table S1). Only adults were included in this study, identified by eruption of the third molar (M3), fusion of the occiput bones and, where available, fusion of epiphyses in post-cranial skeletons.

Three-dimensional models of the skulls were obtained by structured light surface scanning or X-ray CT using several different systems as different authors obtained these (details in electronic supplementary material, table S1). Scan data are stored and publicly available on the online repository Morphosource (project ID: 000344715).

To compare the rabbit cranial diversity with that of the family Leporidae, morphometric data from 301 specimens from 24 species representing all 11 modern genera was taken from existing datasets [47,51]. This sample includes the speciose genera cotton-tail rabbits (*Sylvilagus*), hares or jack-rabbits (*Lepus*), African red-rock hares (*Pronolagus*) and the rare, endangered species such as southeast Asian striped rabbits (*Nesolagus*), southeast Asian hispid hare (*Caprolagus*) and South African riverine rabbit (*Bunolagus*) [25].

### (b) Morphometrics

All analyses were performed in the R Statistical Environment v.4.3.3 [52] using the *geomorph* package v.4.0.7 [53], *stats* package v.4.3.3 [52] and *maps* v.3.4.2 [54].

To characterize the shape of the cranium, we used landmark-based geometric morphometrics. Landmarks were placed on digital models representing the bone in Checkpoint (Stratovan). They were manually placed on the cranium using the ‘landmark’ function: 52 landmarks, including 8 semilandmarks (landmarks with no fixed position along a homologous curve), on the cranium following [47] by different individuals (electronic supplementary material, figure S1, details in table S1). Preliminary analysis of the same specimens digitized by different authors allowed us to ensure the procedures were the same and therefore no variation was due to intraobserver error. Missing landmarks due to bone breakage were estimated using the thin-plate spline method [55] implemented with the ‘estimate.missing’ function in *geomorph*.

The *x*,*y*,*z* coordinates were subjected to Procrustes superimposition [56, p. 376], which removes the effects of translation, rotation and scale. We also considered object symmetry by computing the symmetric component of shape [57], allowing semilandmarks of the cranial roof to slide along their tangent directions in order to minimize bending energy [58], implemented with the ‘bilateral.symmetry’ function in *geomorph*. Centroid size, derived from the landmark coordinates as a measure of size, was computed for each cranium during superimposition.

Principal components analysis of the Procrustes coordinates was used to visualize the morphospace of all 912 specimens, using ‘gm.prcomp’ function in *geomorph*. Multivariate regression was used to assess the amount of cranial shape variation attributed to allometry across all sampled specimens, where the allometric variation here is static allometry, within an age-class. The regression score approach [59] was used to visualize the allometric relationships with natural log-transformed centroid size for all specimens. The predicted line approach [60] was used to visualize an ANCOVA model with an interaction of cranial size and type (wild, domesticated, feral), which allows the identification of different intercepts and slopes among groups. This was implemented with the ‘procD.lm’ function in *geomorph*. To characterize the amount of morphospace occupied by each type, the morphological disparity was calculated as Procrustes variance using the function ‘morphol.disparity’ in *geomorph*. Statistical significance between wild, feral and domesticated groups was assessed through a permutation approach (999 iterations).

Braincase size, as a proxy for brain size, was calculated as the centroid size of landmarks associated with the basicranium and cranial vault (electronic supplementary material, figure S1). Face length was measured as the interlandmark distance between the chiasmatic sulcus (landmark 22) and nasospinale (12) (electronic supplementary material, figure S1). Linear models using ‘lm’ function in the *stats* package were used to evaluate the relationship with log-centroid size.

Linear models were also used to assess the relationship between cranial size and decimal latitude for Northern hemisphere and Southern hemisphere wild and feral rabbits. The samples were plotted on a world map using ‘map’ function of *maps*.

The raw landmark coordinate data of the *Oryctolagus* dataset were combined with the raw landmark data of the 301 leporid specimens, and a Procrustes superimposition and PCA were performed as above to visualize the morphospace. Previously identified principal axes of leporid morphospace [47,51] were manually overlaid onto the combined morphospace to provide an approximate comparison for visualization only. Morphological disparity was calculated for each genus to be numerically compared with *Oryctolagus* as a whole, and only the wild *Oryctolagus*.

## 3. Results

**Figure 1.**
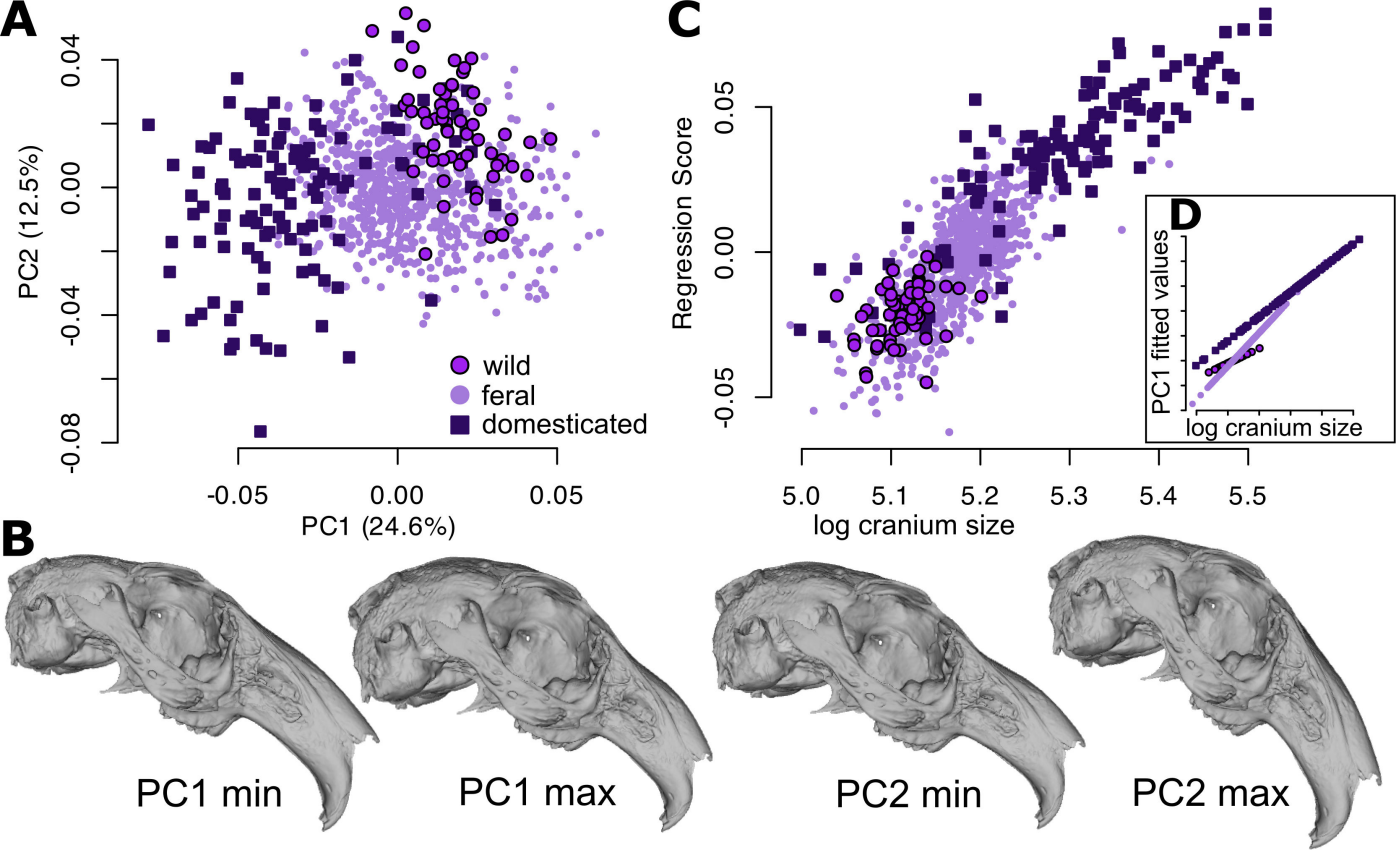
(A) Cranial morphospace represented by the first two principal components (PC) accounting for 37.1% of the variance, showing wild rabbits from the native range, feral and domesticated rabbits. (B) Warped crania representing the shapes of the minima (negative PC scores) and maxima (positive PC scores) of PC1 and 2. See electronic supplementary material, figure S2 for shape change vector graphs of each group. (C) Allometric cranial shape variation is represented by a multivariate regression, where cranial size is the centroid size of the landmarks (here natural log-transformed). Inset: (D) allometric trajectories are different among wild, feral and domesticated species (see electronic supplementary material, figure S3).

Domesticated rabbits (*n* = 121) occupy a much larger region of morphospace than that occupied by wild and feral rabbits with substantial shape differences (figure 1A,B; electronic supplementary material, figure S2), which is attributed in part to their greater size diversity (figure 1C). Overall, they have significantly higher morphological disparity than either feral or wild rabbits (Procrustes variance, PV = 2.8 × 10^–3^, both *p* < 0.001). Feral rabbits (*n* = 732) also occupy a greater amount of the cranial morphospace and are larger in size than wild rabbits (figure 1A,C), but to a lesser extent than domesticated rabbits, while being significantly more varied than wild rabbits (PV = 1.96 × 10^–3^, *p* = 0.009). Some feral rabbits occupy a place in morphospace intermediate to the wild and the domestic rabbits (figure 1A). Wild rabbits (*n* = 59) occupy a narrow region of morphospace (figure 1A), owing in part to a narrow size range (figure 1C). They have significantly less disparity than feral or domesticated rabbits (PV = 1.51 × 10^–3^, *p* = 0.009, *p* < 0.001, respectively). Permutation tests (999 iterations) for morphological disparity allow for assessing statistical significance despite the uneven sample sizes of the three groups.

Allometry is a significant predictor of the observed cranial variation among all sampled specimens, where 16% of the shape variance is attributed to cranial size (R^2^ = 0.16235, F = 185.8562, *p* < 0.001; figure 1C). An analysis of covariance (ANCOVA) modelled with the interaction of cranial size and rabbit type revealed that 2.9% of the variance is attributed to rabbit type (wild, feral or domestic; R^2^ = 0.02913, F = 16.6759, *p* < 0.001) and there is a significant interaction (R^2^ = 0.01711, F = 9.7922, *p* < 0.001), indicating that wild, domesticated, and feral rabbits all have different allometric trajectories describing different shape changes with size (figure 1D; electronic supplementary material, figure S3).

**Figure 2.**
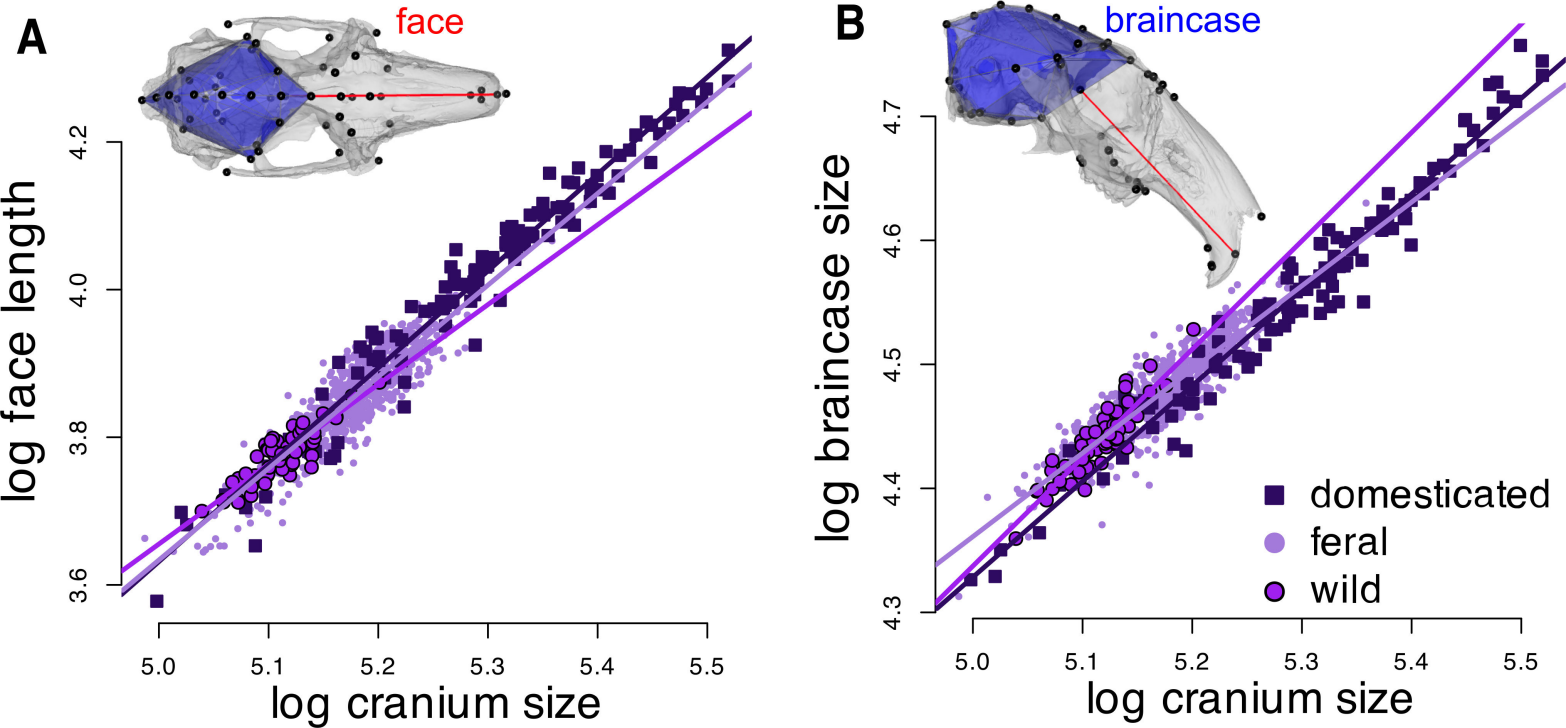
Face and braincase size proportions according to cranial size among wild, feral and domesticated rabbits. (A) Face length (red distance in cranial reconstruction) versus cranial centroid size (natural log-transformed), (B) Braincase size (blue volume in cranial reconstruction) versus cranial centroid size (natural log-transformed), with lines of best fit from separate linear models for each group.

The main axis of cranial shape variation (PC1, 24.6%) is strongly correlated with cranial size (Pearson’s correlation, r = −0.78), and reveals smaller crania with relatively large braincases and short faces (PC1 max), and its inverse pattern for large crania (PC1 min; figure 2B). PC2 (12.5%) is associated with facial tilt (the angle of the face relative to the basicranium), where domesticated individuals demonstrate more variation and in many cases less facial tilt than wild (figure 2B). PC3 (8.1%) has no discernible patterns relating to the three types of rabbit and is not considered further.

Braincase size and face length relative to the whole cranial size differ among wild, domesticated and feral rabbits (figure 2). Domesticated rabbits have relatively longer faces (figure 2A) and proportionally smaller braincase than wild rabbits (figure 2B). In both cases, feral rabbits occupy an intermediate position between wild and domestic.

**Figure 3.**
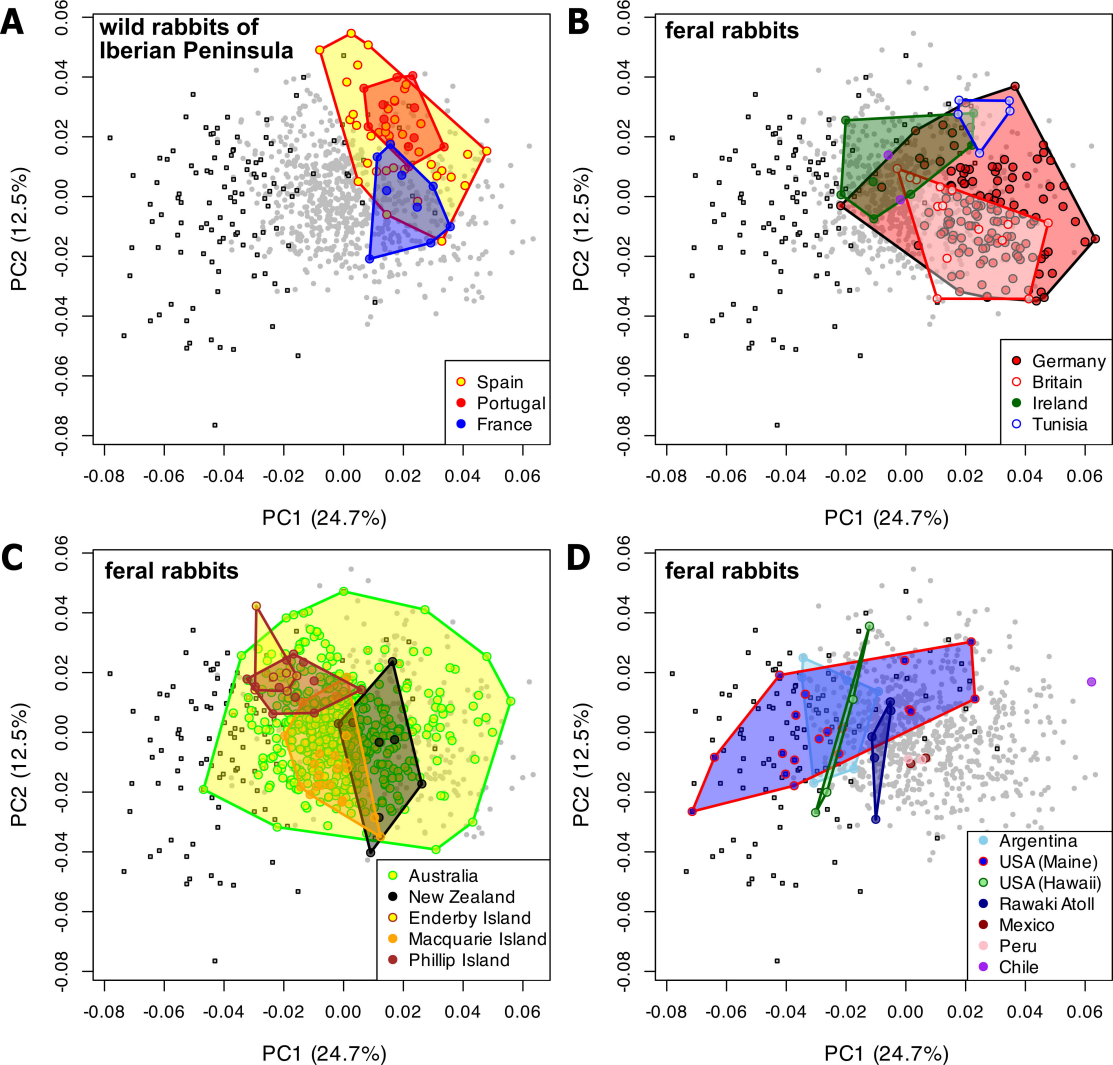
Cranial morphospace represented by the first two principal components (PC) showing wild/feral rabbits (circles) and domesticated (squares), where each panel highlights a different geographical population. (A) Wild rabbits of the Iberian Peninsula, represented by Spain, Portugal, and southwestern France. (B) Feral rabbits from Europe and North Africa. (C) Feral rabbits from Australasian localities. (D) Feral rabbits from other world localities.

Much of the morphological diversity among feral rabbits is partitioned by geographical locality (figure 3; electronic supplementary material, figure S4). Feral rabbits from European and North African populations (figure 3B; electronic supplementary material, figure S4B) occupy some of the same region of the morphospace as the wild rabbits native to the Iberian Peninsula (figure 3A; electronic supplementary material, figure S4A), but occupy more of the morphospace through being larger, especially in Ireland, Germany and Britain (electronic supplementary material, figure S4B). Feral populations of areas further away from the native range occupy a larger proportion of the morphospace, particularly an area unoccupied by wild specimens (figure 3C,D; electronic supplementary material, figure S4C and S4D). Some of the largest rabbits in this dataset are found in Enderby Island (600 km southwest of Dunedin, New Zealand, electronic supplementary material, figure S4C), Phillip Island (near Norfolk Island, ~1470 km east of Brisbane, Australia, electronic supplementary material, figure S4C), and Argentina and USA (Maine and Hawaii Laysan Island; electronic supplementary material, figure S4D).

Given that feral rabbits are generally much larger than wild rabbits (figure 1C), it is necessary to consider Bergmann’s rule, which states that populations of larger body sizes should be found in colder environments. Concomitantly, populations of smaller body sizes will be found in warmer regions. Latitude is a commonly used variable when testing this rule, and our sample covers a range of latitudes in both hemispheres (electronic supplementary material, figure S5A). We found that size variation in the feral and wild rabbits varies among geographical locations but is not strictly correlated with latitude (electronic supplementary material, figure S5B): in the Northern Hemisphere, there is no correlation between rabbit size and latitude (*R*^2^ = 0.00824, *p* = 0.167, slope = 0.0686); in Southern Hemisphere locations, there is a weak negative correlation (*R*^2^ = 0.0317, *p* < 0.001, slope = −0.247). Furthermore, Foster’s rule, where islands are expected to promote gigantism in small animals, is also not supported by these data as many mainland localities have equally large specimens (electronic supplementary material, figure S5C).

**Figure 4.**
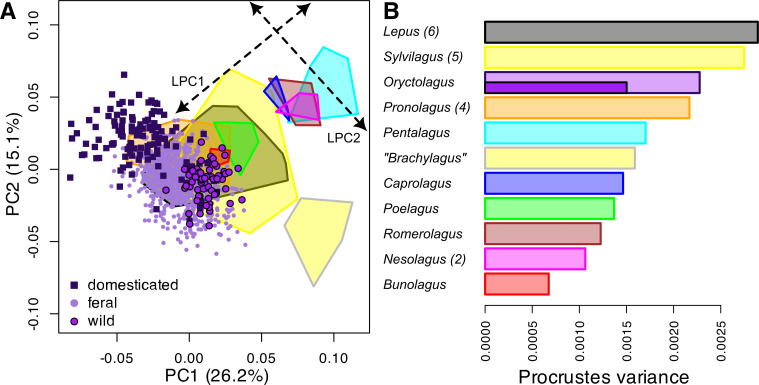
(A) Cranial morphospace of all 11 modern genera of Leporidae (24 species), showing the position of wild/feral (circles) and domesticated (squares) rabbits, and convex hull polygons of genera (colours explained in (B)). Dotted line arrows overlaid demarcate the direction of two principal axes of Leporidae cranial variation (LPC1 and LPC2) as described previously by Kraatz & Sherratt [47]. (B) Morphological disparity (Procrustes variance) of each genus, with the number of species in parentheses when more than one. For *Oryctolagus*, disparity is calculated from all specimens, with disparity for only the wild specimens given as small inset bar.

European rabbit (*Oryctolagus*) cranial diversity occupies a substantial proportion of the morphospace compared to genera of the family Leporidae (figure 4). They appear to have diversified along a trajectory that aligns with the direction of the two principal shape axes previously described for all species of Leporidae, marked as LPC1 and LPCA2 in figure 4A [47,51]. The breadth of variation in *Oryctolagus* (PC2, figure 2A) aligns with LPC1 axis (figure 4A), while the main axis of variation in *Oryctolagus* (PC1, figure 1A) aligns with LPC2, which is an axis describing allometry, where the minimal extent is occupied by the pygmy rabbit *Sylvilagus* (formerly *Brachylagus*) *idahoensis* [61] (figure 4A). However, European rabbits have also expanded into unoccupied regions of the morphospace compared to the Leporidae species shown. Overall, wild, feral and domesticated rabbits—which are members of a single species—occupy almost as much of the morphospace as the two most specious genera (*Lepus* and *Sylvilagus*, comprising six and five species, respectively), and more than the *Pronolagus* genus here comprising four species and all other genera (figure 4B). The wild form of *Oryctolagus*, on the other hand, occupies a portion of the morphospace similar in extent as the other single-species genera (figure 4B).

## 4. Discussion

In the relatively short time that European rabbits have been domesticated and introduced by humans far outside of their natural geographical range of the Iberian Peninsula, they have acquired a large amount of variation in cranial shape and size (figure 1). The magnitude of morphological disparity displayed by our *Oryctolagus* sample is comparable to that found within speciose genera of leporids, which is a consequence of feral and domesticated rabbits diverging into unexplored regions of leporid morphospace (figure 4). However, the main axes of shape diversity within rabbits align with those observed among all leporids [47], endorsing the findings that there are shared patterns of morphological integration in leporids, which facilitate and direct morphological diversity among species and during domestication and feralization [62].

A previous study with a limited sample size of wild and domesticated rabbit specimens presented allometry as a hypothesized pathway to morphological diversity [3]. Our study with a large sampling of feral specimens substantiates the conclusion that rabbit cranial shape variation is driven at least in part by body size diversity (figure 1C,D). Among domesticates, size variation is particularly pronounced, with rabbits typically spanning 1−9 kg (see [3], and references therein). Evolutionary allometry, the correlation of shape with size along branches of the phylogeny, is not statistically significant in Leporidae [51] even though allometric shape changes are a principal axis of leporid cranial shape variation (a result of the distinct shape of the pygmy rabbit, *Sylvilagus* [*Brachylagus*] *idahoensis*) (LPC2, figure 4). In other mammals, on the other hand, allometric scaling is a common pattern observed across different clades (e.g. [63]). In particular, body size scales negatively allometrically with brain (case) size, leading to relatively large brain cases in small mammals (‘Rule of Haller’), and relatively longer faces in many larger mammalian taxa, which is likely a consequence of functional requirements for mastication [reviewed in [64]. Diversifying along an allometric axis in *Oryctolagus* conforms to the hypothesis that size constitutes an ‘evolutionary line of least resistance’ (e.g. [65]).

Domestication and breed formation may result in increased phenotypic diversity in various species, including dogs [2], pigs [5], cattle [6], goats [66], South American camelids [67], horses [7], chickens [8], goldfish [9] and pigeons [4]. While the disparity in domestic rabbits is not at the magnitude seen in domestic dogs, many breeds of dog have also contributed to an expansion in morphospace along macroevolutionary axes of cranial shape variation into regions unexplored by wild canid species [2]. More specifically, the occupation of dogs in the morphospace of Carnivora follows—at least in part—a strong allometric gradient for the craniofacial complex [2], a resulting major component for cranial phenotypic variation also discovered in domestic rabbits. Besides these allometric patterns, other factors might play a role in cranial shape variation in domestics such as breed-specific selection processes and genetic drift (e.g. [6]).

Domesticated rabbits do not fully conform to predicted cranial changes associated with the ‘domestication syndrome’ [11]; they exhibit proportionally longer faces instead of the predicted shorter faces, but they have—as predicted—proportionally smaller braincases (figure 2). This pattern is more in line with a pervasive craniofacial allometry already well-studied in mammals [reviewed in [64]. Brain size decrease in domesticates relative to their wild counterparts—including in rabbits—is among the most consistent patterns in domestication [68]. Indeed, Darwin observed ‘This elongation of the skull relatively to its breadth, I find a universal character, not only with the large lop-eared rabbits, but in all the artificial breeds; as is well seen in the skull of the Angora … As the brain has not increased, the bony case enclosing it has not increased, and this has evidently affected through correlation the breadth of the entire skull from end to end’ [1, pp. 116–117]. Rabbits, thus, seem to be exceptions to the short-faced domestication expectation. As argued by Mitchell *et al.* [64, p. 17] the observed ‘hyperallometric gracilisation’ of the rabbit skull is largely a product of three main factors: (i) moderation of bone resources; (ii) a range of other, well-documented selective advantages arising from an elongate viscerocranium; and (iii) some possible secondary developmental influences of negative braincase and orbit allometry. Further research into the biomechanics of the rabbit skull is needed to address these aspects.

Feral rabbits occupy a size range and portion of the morphospace both intermediate between wild and domestic rabbits, but also in large parts overlapping with them (figure 1). Despite allometric patterns describing some of the disparity, feralization in rabbits is not morphologically predictable if extrapolated from the wild or the domestic stock (figure 1D; electronic supplementary material, figure S3). That feralization in rabbits does not simply lead to a morphological reversal to the wild form has been described previously for an island population [69] but is a novel observation for rabbits more broadly.

The data presented here will prove valuable in gaining a deeper understanding of the extrinsic factors responsible for morphological diversity in widespread feral populations of rabbits. So far, we understand that despite today’s great geographical spread, latitudinal gradients (Bergmann’s rule) do not appear to play a substantial role in the observed diversity (electronic supplementary material, figure S4). Rather, human proximity and/or novel environmental conditions [70–72] in the introduced range might influence this pattern. The greater diversity seen in the cranial characteristics of feral rabbit populations introduced outside their allochthonous ranges, which is by trend more pronounced with increasing distance from these allochthonous range, could be related to changes in evolutionary pressures. Exposure to different environments and predators in introduced ranges may drive rabbit populations to evolve cranial shape traits that help them survive in novel environments as has been shown in other species (e.g. [71,73]). This might include morphologies similar to the autochthonous population, but also specific ones. Alternatively, rabbits may be able to express more trait plasticity when exposed to fewer evolutionary pressures in these new environments (e.g. [74]). In particular, relaxed functional demands in habitats that are free of large predators, such as Australia and New Zealand, might drive body size variation and thus cranial shape variation in introduced rabbits. In addition, drift due to small founder populations might play a role in the observed patterns. The presence of rabbits on multiple islands [29,30] provides an experimental setup to test these predictions.

Among leporids facial tilt has been found to be a prominent axis of cranial shape variation among species (LPC1, figure 4) and is related to the degree of cursoriality [47]; more cursorial species have a more pronounced tilt of the face relative to the basicranium. Whether this locomotory factor also explains the facial tilt variation within *Oryctolagus* (figure 1A) is yet to be determined. We observe that wild rabbits have by trend greater facial tilt than many feral and domesticated specimens (figure 1A,B), which has been historically observed in some domestic rabbits [69]. Therefore, this axis of disparity requires further research to understand if changing modes of locomotion in feral and domestic rabbits, as a consequence of varying selection regimes, results in morphological changes to the skull seen at a macroevolutionary level.

An important caveat to our study is that it is not clear when during their historic, human-induced range expansion throughout Europe, rabbits were domesticated and which stages on the domestication continuum are represented in the rabbit populations introduced to Australasia, the Americas and other parts of the world. A recent genomic study on different populations found that feral rabbits are characterized by a mixture of wild and domestic ancestry [22]. Most rabbits relocated and introduced were ‘meat rabbits’, domesticated and bred for food [25]. Therefore, a categorical classification (‘wild, ‘domestic’ and ‘feral’) within the continuous domestication process is a statistical compromise rather than a definitive and real discretization.

Adding to the difficulty, it is not clear if and to what extent there has been introgression of domestic rabbits into the native ‘wild’ population in the autochthonous range. Therefore, shape variation among the investigated rabbit populations should not be considered as directionally aligned categories, leading from ‘wild’ in the native range to ‘domestic’ to ‘feral’ in the introduced ranges, but rather more fluidly and non-directional, with populations that have been more or less shaped by artificial selection (intentional or not). However, it is clear from the data presented here that there are distinct morphological consequences of domestication and feralization that stand these animals apart from rabbits sourced from the original home range. Further studies could include archaeozoological rabbit remains to further investigate pre-domestication wild morphotypes.

In conclusion, this study has shown that domestication and feralization of *O. cuniculus*, in concert with the colonization of new habitats and environments, has leveraged evolutionary patterns (and probably associated processes) ubiquitous in the leporid clade. Our study adds evidence to the hypothesis that domestication drives increased phenotypic variation expanding the morphospaces with morphologies yet unexplored by their wild counterparts or other species of the family. We also provide new inferences on the consequences of feralization, which in the case of rabbits is not necessarily a return to the wild form. Feral rabbits might exhibit morphologies as seen in domesticated or wild forms, but may also exhibit intermediate ones. Such knowledge provides insights into how novel morphologies can evolve at the macroevolutionary level. Future studies may elucidate the evolutionary, functional and developmental drivers and constraints of the specific cranial shape patterns observed in the rabbit.

## Data Availability

Raw morphometric data and R scripts are available from Figshare [75]. Scan data are stored and publicly available on the online repository Morphosource (project ID: 000344715). Supplementary material is available online [76].

## References

[1] Darwin C. 1868 The variation of animals and plants under domestication. London, UK: John Murray.

[2] Drake AG, Klingenberg CP. 2010 Large‐scale diversification of skull shape in domestic dogs: disparity and modularity. Am. Nat. **175**, 289–301. (10.1086/650372) 20095825

[3] Geiger M, Sánchez‐Villagra MR, Sherratt E. 2022 Cranial shape variation in domestication: a pilot study on the case of rabbits. J. Exp. Zool. Part B **338**, 532–541. (10.1002/jez.b.23171) PMC9804214 35934897

[4] Young NM, Linde-Medina M, Fondon JW, Hallgrímsson B, Marcucio RS. 2017 Craniofacial diversification in the domestic pigeon and the evolution of the avian skull. Nat. Ecol. Evol. **1**, 1–8. (10.1038/s41559-017-0095) 28812673 PMC5559897

[5] Owen J, Dobney K, Evin A, Cucchi T, Larson G, Vidarsdottir US. 2014 The zooarchaeological application of quantifying cranial shape differences in wild boar and domestic pigs (Sus scrofa) using 3D geometric morphometrics. J. Archaeol. Sci. **43**, 159–167. (10.1016/j.jas.2013.12.010)

[6] Veitschegger K, Wilson LA, Nussberger B, Camenisch G, Keller LF, Wroe S, Sánchez-Villagra MR. 2018 Resurrecting Darwin’s Niata - anatomical, biomechanical, genetic, and morphometric studies of morphological novelty in cattle. Sci. Rep. **8**, 1–11. (10.1038/s41598-018-27384-3) 29904085 PMC6002398

[7] Heck L, Wilson LAB, Evin A, Stange M, Sánchez-Villagra MR. 2018 Shape variation and modularity of skull and teeth in domesticated horses and wild equids. Front. Zool. **15**, 14. (10.1186/s12983-018-0258-9) 29713365 PMC5907714

[8] Stange M, Núñez-León D, Sánchez-Villagra MR, Jensen P, Wilson LA. 2018 Morphological variation under domestication: how variable are chickens? R. Soc. Open Sci. **5**, 180993. (10.1098/rsos.180993) 30225085 PMC6124038

[9] Le Verger K, Küng LC, Fabre AC, Schmelzle T, Wegmann A, Sánchez-Villagra MR. 2024 Goldfish phenomics reveals commonalities and a lack of universality in the domestication process for ornamentation. Evol. Lett. **8**, qrae032. (10.1093/evlett/qrae032) PMC11637523 39677575

[10] Wagner F, Ruf I. 2019 Who nose the borzoi? Turbinal skeleton in a dolichocephalic dog breed (Canis lupus familiaris). Mamm. Biol **94**, 106–119. (10.1016/j.mambio.2018.06.005)

[11] Wilkins AS, Wrangham RW, Fitch WT. 2014 The ‘domestication syndrome’ in mammals: a unified explanation based on neural crest cell behavior and genetics. Genetics **197**, 795–808. (10.1534/genetics.114.165423) 25024034 PMC4096361

[12] Lord KA, Larson G, Coppinger RP, Karlsson EK. 2020 The history of farm foxes undermines the animal domestication syndrome. Trends Ecol. Evol. **35**, 125–136. (10.1016/j.tree.2019.10.011) 31810775

[13] Herre W, Röhrs M. 1990 Haustiere–zoologisch gesehen. Heidelberg, Germany: Springer. (10.1007/978-3-642-39394-5)

[14] Sánchez-Villagra MR, Segura V, Geiger M, Heck L, Veitschegger K, Flores D. 2017 On the lack of a universal pattern associated with mammalian domestication: differences in skull growth trajectories across phylogeny. R. Soc. Open Sci. **4**, 170876. (10.1098/rsos.170876) 29134088 PMC5666271

[15] Zeder MA. 2020 Straw foxes: domestication syndrome evaluation comes up short. Trends Ecol. Evol. **35**, 647–649. (10.1016/j.tree.2020.03.001) 32668211

[16] Gering E, Incorvaia D, Henriksen R, Conner J, Getty T, Wright D. 2019 Getting back to nature: feralization in animals and plants. Trends Ecol. Evol. **34**, 1137–1151. (10.1016/j.tree.2019.07.018) 31488326 PMC7479514

[17] Kruska D, Röhrs M. 1974 Comparative--quantitative investigations on brains of feral pigs from the Galapagos Islands and of European domestic pigs. Z. Fur Anat. Und Entwicklungsgeschichte **144**, 61–73. (10.1007/BF00518633) 4851103

[18] Souquet L, Chevret P, Ganem G, Auffray JC, Ledevin R, Agret S, Hautier L, Renaud S. 2019 Back to the wild: does feralization affect the mandible of non-commensal house mice (Mus musculus domesticus)? Biol. J. Linn. Soc. **126**, 471–486. (10.1093/biolinnean/bly218)

[19] Neaux D, Sansalone G, Lecompte F, Haruda A, Schafberg R, Cucchi T. 2020 Examining the effect of feralization on craniomandibular morphology in pigs, Sus scrofa (Artiodactyla: Suidae). Biol. J. Linn. Soc. **131**, 870–879. (10.1093/biolinnean/blaa156)

[20] Pohle AK, Zalewski A, Muturi M, Dullin C, Farková L, Keicher L, Dechmann DKN. 2023 Domestication effect of reduced brain size is reverted when mink become feral. R. Soc. Open Sci. **10**, 230463. (10.1098/rsos.230463) 37416828 PMC10320332

[21] Daniels TJ, Bekoff M. 1989 Population and social biology of free-ranging dogs, Canis familiaris. J. Mammal. **70**, 754–762. (10.2307/1381709)

[22] Andrade P *et al*. 2024 Selection against domestication alleles in introduced rabbit populations. Nat. Ecol. Evol. **8**, 1543–1555. (10.1038/s41559-024-02443-3) 38907020

[23] Irving-Pease EK, Frantz LAF, Sykes N, Callou C, Larson G. 2018 Rabbits and the Specious Origins of Domestication. Trends Ecol. Evol. **33**, 149–152. (10.1016/j.tree.2017.12.009) 29454669

[24] Callou C. 2003 De la garenne au clapier: étude archéozoologique du lapin en europe occidentale. Paris, France: Muséum national d’Histoire naturelle.

[25] Kraatz B *et al*. 2021 Lagomorpha as a model morphological system. Front. Ecol. Evol. **9**, 636402. (10.3389/fevo.2021.636402)

[26] Cooke BD, Fenner F. 2002 Rabbit haemorrhagic disease and the biological control of wild rabbits, Oryctolagus cuniculus. Wildl. Res. **29**, 689–706. (10.1071/WR02010)

[27] Gübelin P, Correa-Cuadros JP, Ávila-Thieme MI, Flores-Benner G, Duclos M, Lima M, Jaksic FM. 2023 European rabbit invasion in a semi-arid ecosystem of Chile: how relevant is its role in food webs? Life **13**, 916. (10.3390/life13040916) 37109445 PMC10144028

[28] Bonino N, Soriguer R. 2009 The invasion of Argentina by the European wild rabbit Oryctolagus cuniculus. Mammal Rev. **39**, 159–166. (10.1111/j.1365-2907.2009.00146.x)

[29] Flux JEC, Fullagar PJ. 1992 World distribution of the rabbit Oryctolagus funiculus on islands. Mammal Rev. **22**, 151–205. (10.1111/j.1365-2907.1992.tb00129.x)

[30] Watson JS. 1961 Feral rabbit populations on pacific islands. Pac. Sci **15**, 591–593.

[31] Lees AC, Bell DJ. 2008 A conservation paradox for the 21st century: the European wild rabbit Oryctolagus cuniculus, an invasive alien and an endangered native species. Mammal Rev. **38**, 304–320. (10.1111/j.1365-2907.2008.00116.x)

[32] Pelletier M. 2019 Morphological diversity of wild rabbit populations: implications for archaeology and palaeontology. Biol. J. Linn. Soc. **128**, 211–224. (10.1093/biolinnean/blz074)

[33] Pelletier M. 2021 Morphological diversity, evolution and biogeography of early Pleistocene rabbits (Genus Oryctolagus). Palaeontology **64**, 817–838. (10.1111/pala.12575)

[34] Falzon I. 2019 Craniometrical studies on the skull of the wild rabbit, Oryctolagus cuniculus (Linnaeus, 1758) (Mammalia Leporidae), in the Maltese archipelago. Biodivers. J. **10**, 269–274. (10.31396/biodiv.jour.2019.10.3.269.274)

[35] Lo Valvo M, La Scala A, Scalisi M. 2014 Biometric characterisation and taxonomic considerations of European rabbit Oryctolagus cuniculus (Linnaeus 1758) in Sicily (Italy). World Rabbit Sci. **22**, 207–209. (10.4995/wrs.2014.1467)

[36] Vigne JD, Biju-Duval C, Soriguer R, Dennebouy N, Monnerot M. 1994 Multiple characterization of a reference population of European rabbit (oryctolagus cuniculus): las lomas (Southern Spain). Pol. Ecol. Stud. 583–596.

[37] Williams CK, Moore RJ. 1989 Phenotypic adaptation and natural-selection in the wild rabbit, Oryctolagus cuniculus, in Australia. J. Anim. Ecol. **58**, 495–507. (10.2307/4844)

[38] Taylor J, Freedman L, Olivier TJ, McCluskey J. 1977 Morphometric differences between Australian wild rabbit populations. Aust. J. Zool. **25**, 721–732.

[39] McCluskey J, Olivier TJ, Freedman L, Hunt E. 1974 Evolutionary divergences between populations of Australian wild rabbits. Nature **249**, 278–279. (10.1038/249278a0) 4833246

[40] Myers K. 1970 The rabbit in Australia. In Proc. Adv. Study. Inst. Dynamics Number Popul, Oosterbeek, pp. 478–506. Purdoc, Wageningen.

[41] Sharples CM, Fa JE, Bell DJ. 1996 Geographical variation in size in the European rabbit Oryctolagus cuniculus (Lagomorpha: Leporidae) in western Europe and North Africa. Zool. J. Linn. Soc. **117**, 141–158. (10.1111/j.1096-3642.1996.tb02153.x)

[42] Davis SJM. 2019 Rabbits and Bergmann’s rule: how cold was Portugal during the last glaciation? Biol. J. Linn. Soc. **128**, 526–549. (10.1093/biolinnean/blz098)

[43] Sanchez-Villagra MR. 2022 The process of animal domestication. Princeton, NJ: Princeton University Press. (10.23943/princeton/9780691217666.001.0001)

[44] Fiorello CV, German RZ. 1997 Heterochrony within species: craniofacial growth in giant, standard, and dwarf rabbits. Evolution **51**, 250–261. (10.2307/2410978) 28568789

[45] Böhmer C, Böhmer E. 2017 Shape variation in the craniomandibular system and prevalence of dental problems in domestic rabbits: a case study in evolutionary veterinary science. Vet. Sci. **4**, 5. (10.3390/vetsci4010005) 29056664 PMC5606619

[46] Bramble DM. 1989 Cranial specialization and locomotor habit in the Lagomorpha. Am. Zool. **29**, 303–317. (10.1093/icb/29.1.303)

[47] Kraatz BP, Sherratt E. 2016 Evolutionary morphology of the rabbit skull. PeerJ **4**, e2453. (10.7717/peerj.2453) 27688967 PMC5036099

[48] Todorov OS, Hird C, Kraatz B, Sherratt E, Hill N, de Sousa AA, Blomberg S, Weisbecker V. 2022 Down a rabbit hole: burrowing behaviour and larger home ranges are related to larger brains in leporids. J. Mamm. Evol. **29**, 957–967. (10.1007/s10914-022-09624-6)

[49] Wood-Bailey AP, Cox PG, Sharp AC. 2022 The evolution of unique cranial traits in leporid lagomorphs. PeerJ **10**, e14414. (10.7717/peerj.14414) 36518283 PMC9744148

[50] Hopkins SSB. 2018 Estimation of body size in fossil mammals. In Methods in paleoecology: reconstructing cenozoic terrestrial environments and ecological communities (eds DA Croft, DF Su, SW Simpson), pp. 7–22. Cham, Switzerland: Springer International Publishing. (10.1007/978-3-319-94265-0_2)

[51] Sherratt E, Kraatz B. 2023 Multilevel analysis of integration and disparity in the mammalian skull. Evolution **77**, 1006–1018. (10.1093/evolut/qpad020) 36775928

[52] R Development Core Team. 2024 R: a language and environment for statistical computing, v.4.4.2. See http://www.R-project.org.

[53] Adams DC, Collyer ML, Kaliontzopoulou A, Baken EK. 2024 geomorph: software for geometric morphometric analyses, v.3.0.7. CRAN. See http://cran.r-project.org/package=geomorph.

[54] Deckmyn A. 2023 Maps: draw geographical maps, v.3.4.0. CRAN. See https://cran.r-project.org/package=maps.

[55] Gunz P, Mitteroecker P, Neubauer S, Weber GW, Bookstein FL. 2009 Principles for the virtual reconstruction of hominin crania. J. Hum. Evol. **57**, 48–62. (10.1016/j.jhevol.2009.04.004) 19482335

[56] Dryden IL, Mardia KV. 1998 Statistical shape analysis. Chichester, UK: Wiley.

[57] Klingenberg CP, Barluenga M, Meyer A. 2002 Shape analysis of symmetric structures: quantifying variation among individuals and asymmetry. Evolution **56**, 1909–1920. (10.1111/j.0014-3820.2002.tb00117.x) 12449478

[58] Gunz P, Mitterocker P, Bookstein FL. 2005 Semilandmarks in three dimensions. In Modern morphometrics in physical anthropology (ed. DE Slice), pp. 73–98. New York, NY: Kluwer Academic/Plenum Publishers.

[59] Drake AG, Klingenberg CP. 2008 The pace of morphological change: historical transformation of skull shape in St Bernard dogs. Proc. R. Soc. B **275**, 71–76. (10.1098/rspb.2007.1169) PMC2562403 17956847

[60] Adams DC, Nistri A. 2010 Ontogenetic convergence and evolution of foot morphology in European cave salamanders (Family: Plethodontidae). BMC Evol. Biol. **10**, 216. (10.1186/1471-2148-10-216) 20637087 PMC2927916

[61] Cano-Sánchez E, Rodríguez-Gómez F, Ruedas LA, Oyama K, León-Paniagua L, Mastretta-Yanes A, Velazquez A. 2022 Using ultraconserved elements to unravel lagomorph phylogenetic relationships. J. Mamm. Evol. **29**, 395–411. (10.1007/s10914-021-09595-0)

[62] Sherratt E, Thomson VA, Lee MSY, Dunstan N, Allen L, Abraham J, Palci A, Ammresh. 2023 Island tiger snakes (Notechis scutatus) gain a ‘head start’ in life: how both phenotypic plasticity and evolution underlie skull shape differences. Evol. Biol. **50**, 111–126. (10.1007/s11692-022-09591-z)

[63] Marcy AE, Guillerme T, Sherratt E, Rowe KC, Phillips MJ, Weisbecker V. 2020 Australian rodents reveal conserved cranial evolutionary allometry across 10 million years of murid evolution. Am. Nat. **196**, 755–768. (10.1086/711398) 33211559

[64] Mitchell DR, Sherratt E, Weisbecker V. 2024 Facing the facts: adaptive trade‐offs along body size ranges determine mammalian craniofacial scaling. Biol. Rev. **99**, 496–524. (10.1111/brv.13032) 38029779

[65] Marroig G, Cheverud J. 2010 Size as a line of least resistance II: direct selection on size or correlated response due to constraints? Evolution **64**, 1470–1488. (10.1111/j.1558-5646.2009.00920.x) 20015239

[66] Balcarcel AM, Geiger M, Sánchez-Villagra MR. 2024 Cranial form differences in goats by breed and domestic status. Sci. Rep. **14**, 917. (10.1038/s41598-023-50357-0) 38195639 PMC10776561

[67] Balcarcel AM, Sánchez-Villagra MR, Segura V, Evin A. 2021 Singular patterns of skull shape and brain size change in the domestication of South American camelids. J. Mammal. **102**, 220–235. (10.1093/jmammal/gyaa135)

[68] Balcarcel AM, Geiger M, Clauss M, Sánchez‐Villagra MR. 2022 The mammalian brain under domestication: discovering patterns after a century of old and new analyses. J. Exp. Zool. Part B **338**, 460–483. (10.1002/jez.b.23105) PMC9787656 34813150

[69] Hückinghaus F. 1965 Craniometrische Untersuchung an verwilderten Hauskaninchen von der Kerguelen. Z. Für Wiss. Zool. **171**, 183–196.

[70] Alberti M, Correa C, Marzluff JM, Hendry AP, Palkovacs EP, Gotanda KM, Hunt VM, Apgar TM, Zhou Y. 2017 Global urban signatures of phenotypic change in animal and plant populations. Proc. Natl Acad. Sci. USA **114**, 8951–8956. (10.1073/pnas.1606034114) 28049817 PMC5576774

[71] Amiot C, Lorvelec O, Mandon‐dalger I, Sardella A, Lequilliec P, Clergeau P. 2007 Rapid morphological divergence of introduced red‐whiskered bulbuls Pycnonotus jocosus in contrasting environments. Ibis **149**, 482–489. (10.1111/j.1474-919X.2007.00671.x)

[72] Wang SP, Althoff DM. 2019 Phenotypic plasticity facilitates initial colonization of a novel environment. Evolution **73**, 303–316. (10.1111/evo.13676) 30618131

[73] Phillips BL, Shine R. 2005 The morphology, and hence impact, of an invasive species (the cane toad, Bufo marinus): changes with time since colonisation. Animal Conservation Forum **8**, 407–413. (10.1017/S1367943005002374)

[74] McCann SM, Kosmala GK, Greenlees MJ, Shine R. 2018 Physiological plasticity in a successful invader: rapid acclimation to cold occurs only in cool-climate populations of cane toads (Rhinella marina). Conserv. Physiol. **6**, cox072. (10.1093/conphys/cox072) 29399360 PMC5786208

[75] Sherratt E *et al*. 2025 Data for: From wild to domestic and in between: how domestication and feralization changed the morphology of rabbits. Figshare. (10.25909/28300055) PMC12212994 40592446

[76] Sherratt E *et al*. 2025 Supplementary material from: From wild to domestic and in between: how domestication and feralization changed the morphology of rabbits. Figshare. (10.6084/m9.figshare.c.7859509) PMC12212994 40592446

